# Modeling anticipated changes in numbers of SARS-CoV-2 infections within communities due to immunization campaigns

**DOI:** 10.12688/gatesopenres.13448.2

**Published:** 2022-09-27

**Authors:** Kurt Frey, Brittany Hagedorn, Kevin A. McCarthy, Raymond Hutubessy, Susan Annemarie Wang

**Affiliations:** 1 Institute for Disease Modeling, Bill & Melinda Gates Foundation, Seattle, WA, 98109, USA; 2 World Health Organization, Geneva, Switzerland

**Keywords:** SARS-CoV-2, agent-based modeling, vaccine delivery

## Abstract

**Background: **As SARS-CoV-2 spread in early 2020, uncertainty about the scope, duration, and impact of the unfolding outbreaks caused numerous countries to interrupt many routine activities, including health services. Because immunization is an essential health service, modeling changes in SARS-CoV-2 infections among communities and health workers due to different vaccination activities was undertaken to understand the risks and to inform approaches to resume services.

**Methods: **Agent-based modeling examined the impact of Supplemental Immunization Activities (SIAs) delivery strategies on SARS-CoV-2 transmission in communities and health workers for six countries capturing various demographic profiles and health system performance: Angola, Ecuador, Lao PDR, Nepal, Pakistan, and Ukraine.

**Results: **Urban, fixed-post SIAs during periods of high SARS-CoV-2 prevalence increased infections within the community by around 28 [range:0-79] per 1000 vaccinations. House-to-house SIAs in mixed urban and rural contexts may import infections into previously naïve communities. Infections are elevated by around 60 [range:0-230] per 1000 vaccinations, but outcomes are sensitive to prevalence in health workers and SIA timing relative to peak.

**Conclusions: **Incremental increases in SARS-CoV-2 infection due to SIAs was small and in proportion to overall prevalence. Younger populations experience lower transmission intensity and fewer excess infections per childhood vaccine delivered. Large rural populations have lower transmission intensity but face a greater risk of introduction of SARS-CoV-2 during an SIA.

## Abbreviations

CMCC    COVID-19 Multi-Model Comparison Collaboration

CSSE    Center for Systems Science and Engineering

DTP3     Third dose diphtheria, tetanus toxoid, and pertussis combined vaccine

EMOD    Epidemiological MODeling software

EPI          Essential Programme on Immunization

FP            Fixed-post

GPEI       Global Polio Eradication Initiative

H2H        House-to-house

HW          Health worker

IPC         Infection prevention and control

LMIC       Low- and middle-income countries

RATR     Relative acquisition and transmission rate

SIA         Supplemental Immunization Activity

VPD         Vaccine Preventable Disease

WHO         World Health Organization

## Introduction

The novel coronavirus SARS-CoV-2 spread globally and became a pandemic in early 2020. In March 2020, the WHO issued interim guidance ^1^ emphasizing the need to prioritize continuity of immunization services wherever they could be conducted safely and advised temporary suspension of mass vaccination campaigns based on the recommendations for physical distancing and the understanding of SARS-CoV-2 transmission. As a result of the pandemic, many countries postponed or cancelled planned SIAs in 2020 against polio ^2^, measles ^3^, cholera, yellow fever, and other VPDs ^4^.

SIA delay was driven by concerns over risks of SARS-CoV-2 transmission via SIAs, but there are also health risks, particularly to vulnerable populations, of delay ^5^. In addition, health systems weakened by the COVID-19 pandemic are unable to keep up with day-to-day healthcare needs ^6^, which may leave those who are affected by non-COVID-19 illness more vulnerable to morbidity and mortality. Many countries reconsidered earlier choices to postpone SIAs, rescheduling them to occur during the ongoing pandemic ^7– 9^. The risks of SARS-CoV-2 transmission need to be balanced with the benefits of an SIA. To this end, the WHO published a framework to assist countries with making decisions regarding whether to implement preventive mass vaccination campaigns ^10^.

Routine immunization coverage has also been affected. Reductions in coverage have been documented in communities such as Karachi, Pakistan ^11^ and globally, the WHO reports that more than half of countries with available data had moderate or severe disruptions to immunization services ^12^. The reasons are attributable to a variety of factors, including diversion of health workers to COVID-19 response, lockdowns preventing transportation or community movement, and subsequent reduction in numbers of families seeking immunization for their children, reduced numbers of immunization days in clinic, and less frequent outreach services being offered.

The purpose of the study was to estimate, using one country from each WHO region and spanning a range of COVID-19 disease burden settings, the anticipated changes in numbers of SARS-CoV-2 infections in communities due to immunization campaigns.

## Methods

### Example country selection

We selected six countries representing upper-middle, lower-middle, and low-income contexts; they included priority countries for the Essential Programme on Immunization (EPI) and for the Global Polio Eradication Initiative (GPEI). A country was chosen from each of the six WHO regions. We compared countries across six indicators: percentage coverage of DTP3, number of nurses per thousand total population, percentage of population under 15 years old, human development index, percentage of population living in a rural setting, and percentage of population living in a slum or informal settlement. Selected countries represent different demographics, social structure, overall economic development, and health system strength, to span the range of indicator values. Explanations of indicators, data sources, and 2019 values for other LMIC countries can be found in the *Extended data* ^13^.

### Transmission modeling

Forecasts were generated using EMOD, an individual-based disease modeling platform ^14^ that has been reviewed by the COVID-19 Multi-Model Comparison Collaboration (CMCC) ^15^. Additional details can be found in the *Extended data* ^13^. Simulations were intended to represent SARS-CoV-2 progression in the chosen contexts and parameter values used appropriate for the respective countries.

Infections were represented by a latent period followed by an infectious period. The progression of disease within each person was stochastically variable in duration. At the end of the infectious period, the person is given total immunity from subsequent infection, as was the case early in the pandemic when these questions were being considered. Immunity is assumed to not wane over the course of the simulation. Parameters correspond to a median generation interval of about 6 days. This interval is not specified as an input parameter but is observable in the simulation results and is a consequence of the epidemiological parameters that were input.

A total population of 1M people was used for each simulation. Each simulated person is assigned to an age cohort according to the demographics of the simulated country. Contact rates between simulated persons in the model are stratified across four routes (school, home, work, and community) and sixteen age groups (5-year age groups up to 75 years old, and one age group for those 75+ years old) using published model estimates ^16^, and by risk levels (low, medium, and high). Risk levels provide additional variance within age group without altering mean contact rates.

The baseline distancing scenario for each country assumes school closures, reduced work contacts, and restricted community gatherings (contact rates for school, work, and community are reduced to 0%, 50%, and 75%, respectively, from the values in 17). For all distancing policies involving a reduction in work contacts or school contacts, twenty percent of the reduced contacts were redistributed to the home route to reflect extra time spent in the home. No community contacts were redistributed to the home route.

For each country, the model was used to fit a most-likely R _0_ value and case reporting rate to match reported case counts during the initial outbreak period; case count data were obtained from the COVID-19 Data Repository by the Center for Systems Science and Engineering (CSSE) at Johns Hopkins University < https://github.com/CSSEGISandData/COVID-19> ^18^. Summary data are included in Table 1. Additional details on infectivity calibration can be found in the *Extended data* ^13^. The indicated value for R _0_ in Table 1 does not account for distancing policy and variable susceptibility with age. Contact fractions by route in Table 1 are input parameters from published model estimates ^16^; the R _0_ values were estimated in this study.

**Table 1.** Baseline scenario contact rate fraction by route and estimated base reproductive number (R _0_).

Country	Contact Rate Fraction by Route				R _0_
	Home	School	Work	Community	
Angola	0.188	0.264	0.071	0.477	3.6
Ecuador	0.223	0.211	0.137	0.429	3.2
Lao PDR	0.195	0.222	0.067	0.516	3.0
Nepal	0.175	0.242	0.135	0.449	3.2
Pakistan	0.188	0.251	0.043	0.518	3.6
Ukraine	0.256	0.112	0.277	0.356	3.0

Reduced susceptibility among children is a significant unknown. Several publications ^19– 24^ suggest that the under-15-year-old cohort acquires and transmits SARS-CoV-2 infections at a lower rate than the general population. This model incorporates a reduction in childhood acquisition of about 55% and childhood transmission of 15%, which has a substantial impact on transmission intensity, reducing the total attack rate and slowing the speed of the outbreak. These estimates are only appropriate to the ancestral SARS-CoV-2 strain examined in this work and may not be relevant to any of the variant lineages. Details on the effect of reduced childhood susceptibility can be found in the *Extended data* ^13^.

Connectivity and migration between city centers, peri-urban and rural communities is also poorly documented in many LMICs. In our spatial model, we assume a single large population center, with the sizes of the other population centers (when present) distributed exponentially. These other population centers represent more rural locations and have a minimum population of 100 simulated persons (total simulation population is one million). The percentage of the population in the large population center is equal to the urban fraction of the country; urban fractions are calculated based on the rural fractions, assuming the urban and rural fractions sum to unity. A network of individual mobility between all population centers was based on the distance between and size of population centers. Modeling the outbreak of SARS-CoV-2 using this distributed community connectivity results in a slower growing and extended outbreak.

The ‘urban’ base case is representative of a single major population center without the network of rural locations, while the ‘urban-rural’ base case is representative of a major population center with surrounding rural locations. No simulations examine a rural-only setting. A rural-only setting would consist of a network of small populations without any single major center, and have outcomes dominated by the timing of disease introduction.

Individual importations often do not result in community transmission. Simulations use a low, constant rate of importation into urban centers (from an unspecified external source) to ensure that an outbreak occurs in urban locations. Rural locations do not include this external importation pressure, so rural locations are not guaranteed to experience an outbreak during a simulation. Outcomes for rural-only simulations would reflect this randomness of importations and would not be expected to provide useful insights.

**Figure 1.**
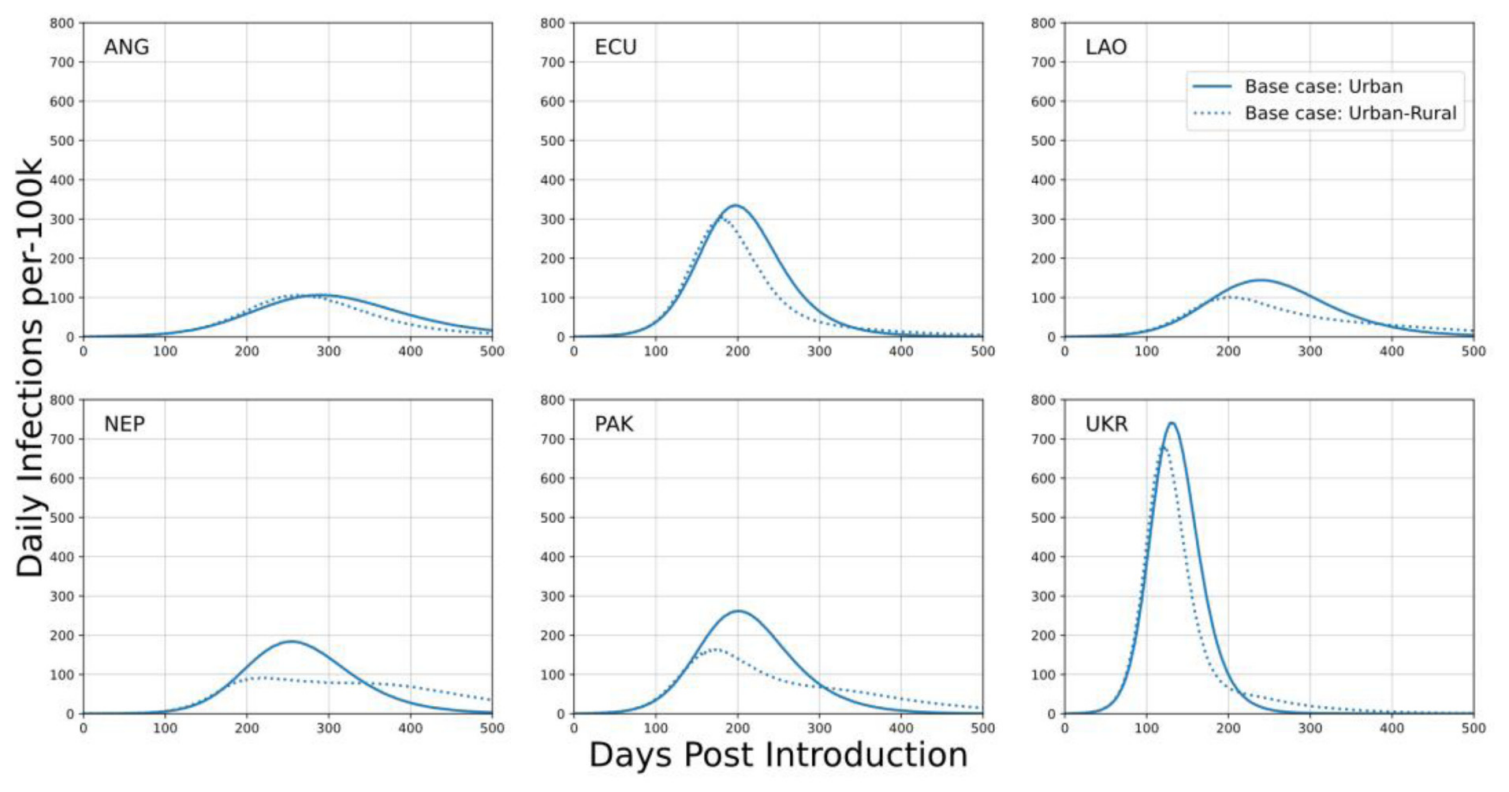
Daily infection trajectories per 100k population for the ‘urban’ base case and for the ‘urban-rural’ base case. In the ‘urban-rural’ base case, the urban fraction of the population is in the largest population center and the remainder of the population is distributed across smaller population centers. The x-axis describes the number of days post SARS-CoV-2 introduction to the community. Baseline scenarios for each country assume school closures, reduced work contacts, and restricted community gatherings.

Both types of base case depicted in Figure 1 are used when presenting results for this study.

Outcomes depicted in Figure 1 (and throughout) are trajectories of mean behavior based on ensembles of 1000 simulations. Timeseries are depicted with respect to ‘days-post-introduction’; SARS-CoV-2 introduction to the community occurs at day-zero on this axis. No adaptive distancing policy is included in these scenarios. For instance, an outbreak as acute as depicted for the Ukraine setting would be expected to result in significant self-modification of behavior, which was not included or examined in this study.

**Figure 2.**
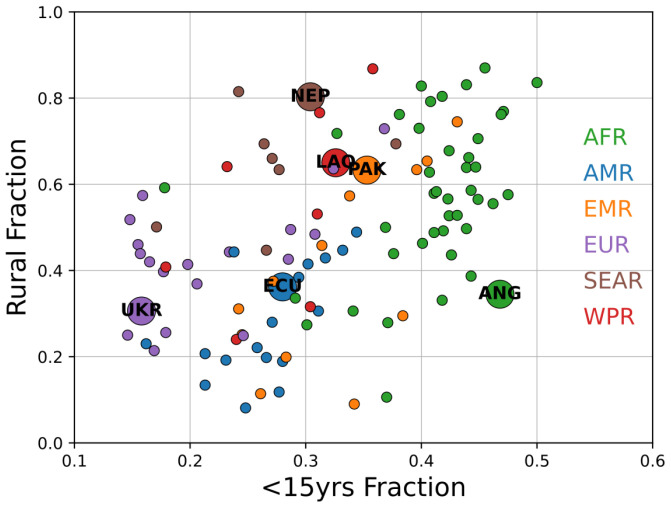
Variation in population fraction younger than 15yrs and rural population fraction in low- and middle-income countries for all six WHO regions. Representative country contexts examined in this study are annotated using larger markers. Size is only intended to highlight marker location.

These scenarios are illustrative of a wide range of potential outcomes, principally depending on the level of urbanization and shape of the population pyramid. Countries were selected as archetype contexts; the range of this variation is depicted in Figure 2 for all LMICs in the six WHO regions. 

### Delivery scenarios

Base case scenarios do not attempt to quantify pre-pandemic levels of routine immunization services or reductions in those services that occurred due to social policy responses at the start of the pandemic. All the scenarios described below are incremental to the base case and the impact of each is calculated as the net difference between the delivery scenario described and this base case value.

Fixed-post (FP) SIAs were reflected in the model by adjusting the contact rates among different age cohorts for seven days, to reflect the community coming together to a central location and having some level of social interaction as well as travel. This was represented by a 50% increase in contacts within the under-5 cohort (children), a 50% increase in contacts among individuals in the 20–35-year-old cohort (their caregivers), and a 200% increase in contacts between the two groups. This scenario approximates a fixed-post SIA with a single-antigen delivered to children, during which vaccination is provided by a health worker who has brief interactions with each child and caregiver. In these scenarios, the increase in community contacts among and between the target population and their caregivers is the primary cause of additional SARS-CoV-2 infections.

House-to-house (H2H) SIAs were reflected in the model by adjusting the interaction rates between health workers and the general population, to reflect the process by which a vaccinator moves from one house to the next administering vaccinations. No changes were made to general community contacts rates with each other. This implies that the children and their caregivers continue to abide by general distancing practices and no additional travel would be required.

To reflect house-to-house SIAs in mixed urban-rural scenarios, health workers were moved from the urban center to smaller communities. For these scenarios, vaccination outreach incorporates many more occurrences of long-distance travel than are present in the baseline mobility structure. In these house-to-house SIAs in rural locations, the potential for health workers to unintentionally introduce SARS-CoV-2 into communities not currently undergoing transmission is the primary cause of additional infections, which then cascade to further infections in the community.

In a typical measles SIA, a vaccinator is expected to deliver between 100–150 vaccinations per day in urban settings and 75–100 per day in more rural areas ^25^. All scenario results report SARS-CoV-2 infections using a per-population basis (e.g., per-100k). Outcomes for fixed-post SIAs incorporate a fractional increase in the number of contacts among the target population and care givers, which accounts for the difference in target population sizes between the contexts. Outcomes for house-to-house SIAs account for the difference in target population sizes by scaling the number of health workers used by the size of the target population. SIA durations and frequency were not varied based on context; however, variations in timing independent of context were examined for sensitivity purposes.

Routine outreach was implemented similarly to a fixed-post SIA, with both children and adults experiencing a 20% increase in intra-community contact rates. However, in the case of outreach, the health worker was expected to interact with both adults and children, since outreach events are intended to serve a wider population. They are also held periodically and consistently; for these purposes we assumed three days per month for a period of three months, with a cumulative 15% of the target population receiving health services.

### Sensitivity analyses

Timing of the vaccination delivery, including scenarios where the delivery occurred prior to peak, near the peak, or after the peak of the SARS-CoV-2 outbreak, was examined as part of the sensitivity analysis.

Impact of infections within health worker populations were varied by simultaneously adjusting the acquisition and transmission rates of health workers; these variations were intended to represent the application of infection prevention and control (IPC) measures. Modifications affected both 1) the acquisition of SARS-CoV2 by the health worker if susceptible and 2) the transmission of SARS-CoV-2 by the health worker if infectious. Levels examined include relative acquisition and transmission rates (RATRs) of 20x, 15x, 10x, 5x, and 1x; levels are for health workers with respect to non-health worker individuals of similar ages. Effectively, these RATRs imply that healthcare workers are always at or above the level of risk of the general population. It is likely HW acquisition and transmission is asymmetric; onward transmission may be more strongly moderated than acquisition based on factors both controllable by health workers (e.g., mask wearing) and structural (e.g., occupational risk). Symmetry has been assumed in this model for simplicity.

The health worker cohort persists for the entire duration of the simulation, the RATRs in this cohort do not change during the vaccine delivery scenarios. Health workers are re-allocated to COVID-19 related tasks (SIAs) during task implementation periods but are assumed to be involved in other health-related activities during other periods. Health worker contact patterns did not follow the age structured matrix used for other groups, see additional file 1 for details. Relative acquisition and transmission rates should be interpreted as an input that controls the overall attack rate of the HW cohort; they capture the aggregate effect of IPC measures and do not correspond to or attempt to quantify the effect of any specific implementation of IPC (e.g., mask wearing or distancing).

## Results

### Routine outreach and fixed-post scenarios

Time to peak SARS-CoV-2 incidence varied by country. For comparability across contexts, vaccination events were timed with respect to time to peak SARS-CoV-2 incidence for the urban setting. These timings can be found in the *Extended data* ^13^. Routine outreach scenarios did not result in outcomes different from the base case in either the urban only or the urban-rural settings. Mean trajectories were reproduced to within available precision.

**Figure 3.**
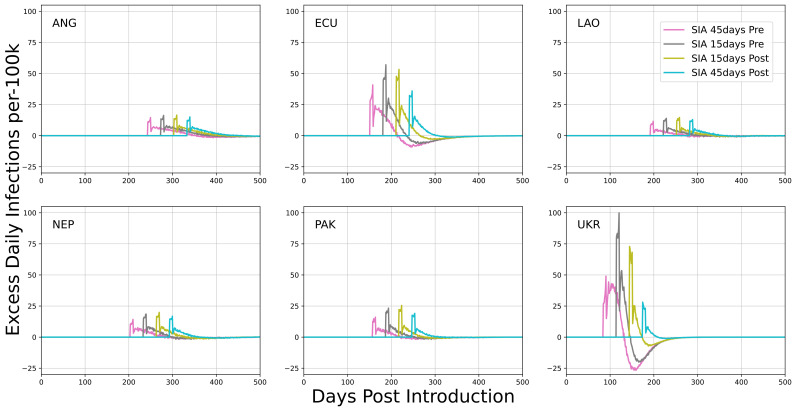
Mean excess daily SARS-CoV-2 infections per 100k individuals in each country examining fixed-post SIAs in urban simulations. Scheduling of SIAs is with respect to the time of peak incidence. The x-axis describes the number of days post SARS-CoV-2 introduction to the community.

Urban, fixed-post SIAs were implemented with respect to the time of SARS-CoV-2 peak incidence. Scenarios examined implementation of a single event 45 days before, 15 days before, 15 days after, or 45 days after peak incidence. Simulated outcomes in Figure 3 implemented each fixed-post SIA independently.

### Impact of relative acquisition/transmission rates in health workers on transmission

Relative acquisition and transmission rates in health workers affect both the overall epidemic for the general population and the attack rate among health workers themselves. The 10x rate in health workers slightly reduces the time to peak incidence for the epidemic relative to the 1x rate, although this change tends to be small. Health workers are a small fraction of the total population (0.1%), but their contacts with vulnerable populations tend to give this cohort an outsized impact for its total size. In scenarios where health workers acquire and transmit infections at the 1x rate, the overall attack rate among health workers is reduced by between 40 to 63 percentage points, compared to scenarios where health workers acquire at the 10x rate (see Table 2).

**Table 2.** Overall SARS-CoV-2 attack rate among health workers (HW) for the two-year period of simulation when varying the relative acquisition/transmission rate (RATR) for the health worker cohort.

Country	Attack Rate (%) in Heath Workers for the Urban Setting				
	HW RATR 20x	HW RATR 15x	HW RATR 10x	HW RATR 5x	HW RATR 1x
Angola	91	86	75	51	13
Ecuador	99	99	97	86	34
Lao PDR	81	75	63	42	11
Nepal	83	80	77	65	24
Pakistan	88	83	71	48	13
Ukraine	99	99	99	98	59

Simulations are focused on disease transmission, and do not address the morbidity or mortality effects that may arise from a depletion of health workers availability due to COVID-19, which was documented in West Africa after the Ebola outbreak of 2014 ^26^. The impact of health worker infections on the progress of the overall epidemic in an urban setting is low or negligible for most contexts.

Acquisition and transmission rates in the health worker cohort are inputs, and overall attack rates for health workers are strongly influenced by those inputs. Contact rates and patterns occurring outside of vaccination activities represent continuing healthcare activities undertaken by health workers. Results presented in Table 2 are the mean attack rates for health workers and in-part reflect the overall force of infection during the epidemic.

### House-to-house scenarios

House-to-house SIAs in urban environments that occur around peak SARS-CoV-2 incidence did not have a measurable impact on infection rates. In these scenarios, health worker contact patterns and rates were reconfigured for the period of the SIA, but this reconfiguration did not result in an elevated number of infections. Ongoing transmission in the urban environment was the primary driver of infections in these house-to-house simulations. It is likely that the vaccination activities did cause additional SARS-CoV-2 infections, but not at a level that was distinguishable from expected base case transmission levels.

An important juxtaposition of this outcome is for house-to-house SIAs in mixed urban and rural environments. For the mixed urban-rural environments, urban health workers were used to systematically visit rural locations for vaccination activities. In these simulations, the SIA was again timed to coincide with peak urban incidence. This delivery method can introduce the virus to locations not experiencing community transmission at the time of the SIA; it is also sensitive to infections among health workers. 

**Figure 4.**
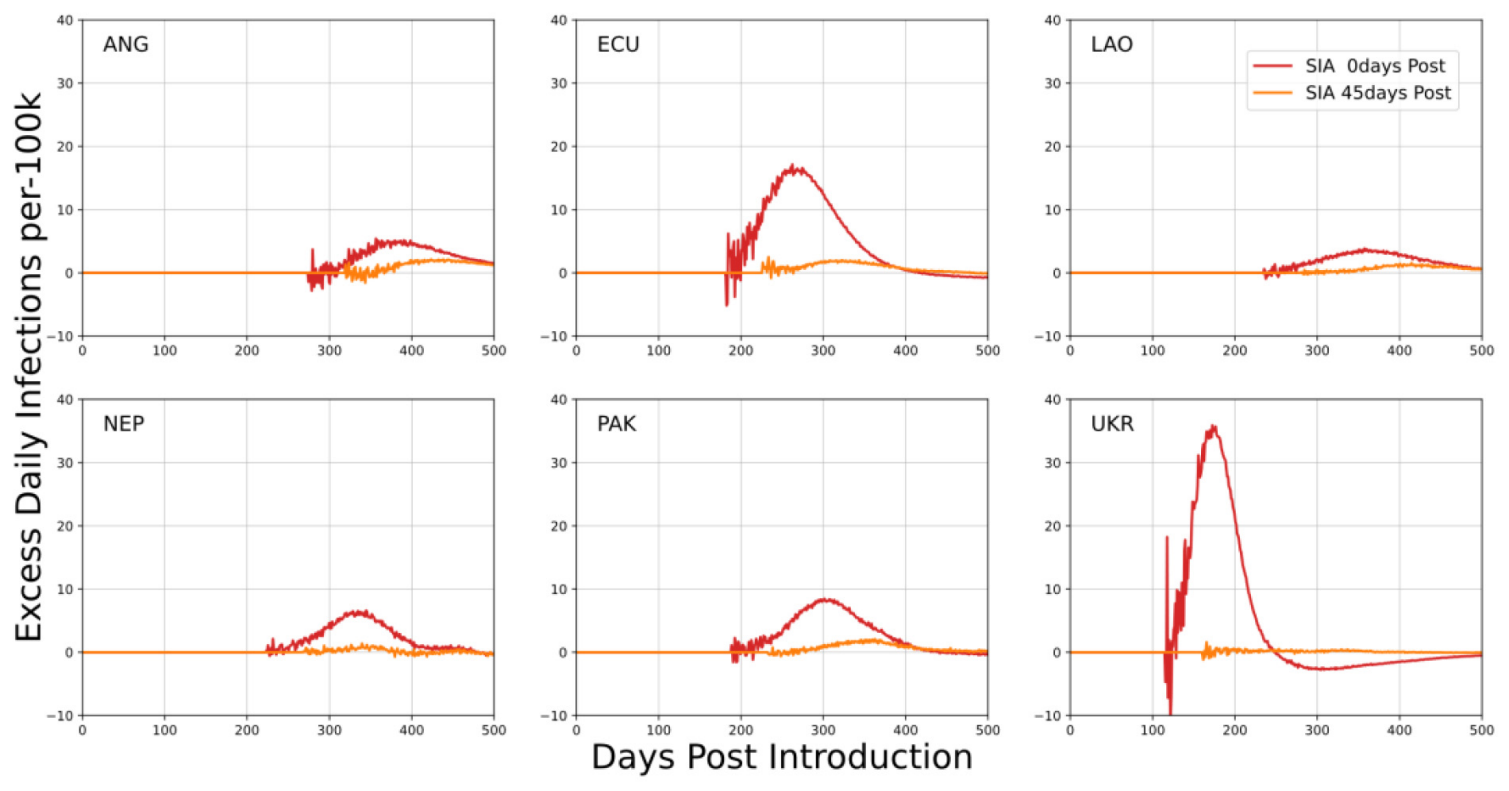
Mean excess daily SARS-CoV-2 infections per 100k individuals in each country examining house-to-house SIAs in mixed urban-rural simulations. Scheduling of SIAs is with respect to the time of peak incidence. The x-axis describes the number of days post SARS-CoV-2 introduction to the community.

Figure 4 depicts the expected increases in infection rates due to such an SIA. Both the level of prevalence and degree of urbanization were contributing factors to the increase in infection rates. Low levels of urbanization corresponded to a greater number of potentially naïve communities at the time of the SIA; higher prevalence at the time of the SIA increased the likelihood of a HW being infected at the time of the SIA and potentially being the cause of a new introduction.

Reducing infections in health workers can mitigate the risk of introduction to naïve communities. Adjusting SIA timing so that it occurs a month or more away from peak incidence also reduced the risk of introductions. Outcomes for these simulations suggest elevated risk only when current prevalence is high and HWs have a greater probability of being infected than the general community. In a high connectivity environment, the increased number of infections associated with an SIA is in proportion to the increased contact rate during the SIA, while with intermediate connectivity (i.e., urban-rural), there is a possibility of non-linear effects.

### Summary

Statistics for urban, fixed-post SIA and rural, house-to-house SIA implementation are summarized in Table 3 and represent the main results of this study. Excess infections due to fixed-post SIAs were largely insensitive to relative acquisition and transmission risk of health workers. Marginal increases in infections in these scenarios were driven by increased community mixing at fixed-posts and not specific interactions with health workers, like how large gatherings for other purposes would be expected to increase transmission.

**Table 3.** Expected excess SARS-CoV-2 infections and infections-per-vaccination, under varying scenarios of relative acquisition and transmission risk (RATR) for health workers (HW) when SIA is undertaken at time of peak COVID-19 incidence. Bracketed ranges span mean outcomes for the six countries.

Delivery method	HW RATR	Excess community infections per 100k total population		Excess community infections per 1k vaccinations	
Fixed-post SIA: urban	20x	310	[0, 550]	27	[0, 75]
	15x	310	[0, 610]	28	[0, 78]
	10x	250	[0, 530]	23	[0, 76]
	5x	230	[0, 560]	22	[0, 77]
	1x	300	[0, 590]	28	[0, 79]
House-to-house SIA: urban-rural	20x	400	[0, 970]	64	[0, 140]
	15x	680	[0, 950]	67	[0, 170]
	10x	520	[0, 1300]	60	[0, 230]
	5x	460	[0, 810]	54	[0, 135]
	1x	120	[0, 410]	17	[0, 75]

All fixed-post scenarios include zero excess infections as a potential outcome. These null results suggest that while the infection rate increases for the duration of the SIA, the overall attack rate for the epidemic is not always substantially affected.

Outcomes for each country demonstrate that marginal increases in infection tend to be small and in proportion to overall prevalence. Angola, Ecuador, and Ukraine are mostly urban with progressively older populations, and baseline transmission intensity increases with mean population age. Pakistan, the Lao PDR, and Nepal are mostly rural, and decreasing mean urban population fraction tends to reduce the severity of initial outbreak along with prolonging the overall duration of outbreak.

Summary statistics in Table 3 are based on the mean outcomes for the scenarios examined in this study, which are biased toward larger excess values because SIA implementation was timed to occur around peak incidence. Countries are expected to demonstrate significant sub-national heterogeneity in epidemic trajectories due to regionally differing times of first introduction. Nationally implemented SIAs would be expected to sample multiple different locations along the trajectories described in this study, resulting in fewer excess infections than in Table 3.

## Discussion

This study helps decision makers in LMICs in understanding the risk-benefit tradeoff of proceeding with immunization services during the pandemic through stylized modeled simulations. There is potentially a large asymmetry in suspending SIAs for diseases like measles and polio: minor increases in SARS-CoV-2 infections averted, but consequently large increases in other vaccine preventable diseases. The modeling approach presented here is a general one and applicable to other emergent infections of pandemic potential, although great care would be necessary selecting pathogen appropriate inputs. SARS-CoV-2 transmission is less intense in young populations, which strongly influences these outcomes.

All these scenarios focus on the increase in SARS-CoV-2 infections to health workers and the community due to vaccination activities, and do not describe the COVID-19 disease burden specifically. While the benefits of vaccination accrue primarily to vaccine recipients (here, the <5yr cohort), the COVID-19 burden will fall most heavily on the aged (>50yr cohort) ^17^. Balancing this asymmetry between risks and benefits across age cohorts is not addressed in this study; the primary outcome is estimating the plausible range of incremental SARS-CoV-2 infections due to SIAs.

Routine outreach scenarios would be expected to lead to some number of additional infections because of the model structure that represents them as an increase in total contacts during the outbreak. The rate of contacts present in the base case as typical behavior makes this increase sufficiently low as to be not distinguishable from zero.

Urban, fixed-post SIAs tended to have a low impact on the ongoing epidemic. Average outcomes were about 20 to 30 additional SARS-CoV-2 infections per 1000 vaccinations. This quantity is biased upward because scenarios examined were in proximity to epidemic peak, which is not an outcome that would be expected to occur during implementation of a nationwide SIA. The model does not include a responsive mechanism for individual behavior with respect to total prevalence and it is likely individuals would self-modify behavior to mitigate acute peaks of the sort represented here. Values are appropriately interpreted as a conservative / high estimate of what would be observed in practice. Additionally, overall attack rates for the entire epidemic were not strongly affected by this SIA implementation. In several scenarios, the change in attack rate was not significantly different from zero even though the rate of infections during the SIA period was elevated. This outcome suggests that infections during the SIA were displacing infections that likely would have occurred later, marginally accelerating the epidemic but not affecting its outcome. This tendency to accelerate the epidemic was most pronounced for interventions occurring before peak incidence. For all fixed-post implementations, excess infections were strongly correlated with prevalence at the time of implementation.

Outcomes describing single-antigen vaccinations are likely extensible to multi-antigen SIAs. Multi-antigen SIAs would be characterized by longer periods of interaction between the health worker and individual receiving vaccination, and an elevated probability of transmission during that interaction. A much larger consideration is the probability that a participant in that interaction is infectious, which is a consequence of prevalence and IPC measures at the time of the intervention.

House-to-house SIAs in mixed urban-rural contexts have the potential to import infections to previously naïve communities. This risk is a consequence of using health workers from urban locations that may be infectious at the time of the SIA.

Reducing prevalence among HWs largely eliminates the scenario where communities (such as rural or semi-isolated populations) will see the first introduction of virus during a SIA. Prevalence also strongly affects the importation risk because of the likelihood that a health worker may be exposed prior to rural travel. Marginal increases in infections in these scenarios represented an increase in mean epidemic attack rate and not an acceleration of the outbreak. Increased mobility correlated with greater importation risk; local vaccination staff should be used wherever possible. In all scenarios, attack rates in health worker populations emphasize the need for protection.

Increased infection experienced by health workers was not examined as a primary outcome in this study. Scenarios examined the potential effects on community infection rates because of varying levels of prevalence among health workers, but not the effect of the SIAs themselves on infections in health workers. Additional work in this area is needed.

Urbanization and age-structure metrics depicted in Figure 2 are the primary covariates examined for this study, and extensions of these results to other countries based on those dimensions provide the greatest inferential power. However, in all simulations the incremental infection due to vaccination activities was small and in proportion to overall prevalence. Urbanization and age structure can help inform expected levels of prevalence of SARS-CoV-2 during an outbreak, but those prevalence levels themselves are the most important guide for SIA implementation; forecasts on the timescale required for SIA planning are not currently achievable. Outcomes in Table 3 should guide expectations for increased infections with respect to peak prevalence, and those measures used to inform the decision on proceeding with a SIA.

## Data Availability

Open Science Framework: Modeling anticipated changes in numbers of SARS-CoV-2 infections within communities due to immunization campaigns, https://doi.org/10.17605/OSF.IO/C3DXR ^13^. This project contains the simulation data. Summary data generated or analyzed during this study are included in the article. Open Science Framework: Modeling anticipated changes in numbers of SARS-CoV-2 infections within communities due to immunization campaigns, https://doi.org/10.17605/OSF.IO/C3DXR ^13^. This project contains the following extended data: Detailed methods for the epidemiological model. Detailed methods for country specific parameters. Scenario results and confidence intervals. Country-level index values. Sensitivity to latent period. Time to peck incidence. Uncertainty analysis Outcomes for multiple fixed-post SIAs. Outcomes for routine outreach. Outcomes in absolute terms. Interpretation of RATR Data are available under the terms of the Creative Commons Attribution 4.0 International license (CC-BY 4.0).
