# Supplementary material for: Modeling anticipated changes in numbers of SARS-CoV-2 infections within communities due to immunization campaigns
Source: Gates Open Res. 2022 Sep 27;6:7. Originally published 2022 Feb 9. [Version 2] doi: 10.12688/gatesopenres.13448.2 (PMC9576906; doi:10.12688/gatesopenres.13448.2)
Supplement: Peer review history

# Peer review: Reviewer response for version 2

**DOI:** 10.21956/gatesopenres.15302.r32615

**Published:** 2022-10-17

**Authors:** Sylvia K. Ofori

**Affiliations:** 1 Department of Biostatistics, Epidemiology and Environmental Health Sciences, Jiann-Ping Hsu College of Public Health, Georgia Southern University, Statesboro, GA, USA

The authors have adequately addressed my comments.

Is the work clearly and accurately presented and does it cite the current literature?

Partly

If applicable, is the statistical analysis and its interpretation appropriate?

Not applicable

Are all the source data underlying the results available to ensure full reproducibility?

Yes

Is the study design appropriate and is the work technically sound?

Yes

Are the conclusions drawn adequately supported by the results?

Partly

Are sufficient details of methods and analysis provided to allow replication by others?

Partly

Reviewer Expertise:

Infectious disease epidemiology and modeling

I confirm that I have read this submission and believe that I have an appropriate level of expertise to confirm that it is of an acceptable scientific standard.

# Peer review: Reviewer response for version 2

**DOI:** 10.21956/gatesopenres.15302.r32616

**Published:** 2022-10-04

**Authors:** Sabina Marchetti

**Affiliations:** 1 Bank of Italy, Rome, Italy

I appreciate the responses and integrations provided by the authors. All main comments were fairly addressed.

Is the work clearly and accurately presented and does it cite the current literature?

Partly

If applicable, is the statistical analysis and its interpretation appropriate?

Not applicable

Are all the source data underlying the results available to ensure full reproducibility?

Yes

Is the study design appropriate and is the work technically sound?

Partly

Are the conclusions drawn adequately supported by the results?

No

Are sufficient details of methods and analysis provided to allow replication by others?

No

Reviewer Expertise:

Mathematical modeling of infectious diseases; Statistics

I confirm that I have read this submission and believe that I have an appropriate level of expertise to confirm that it is of an acceptable scientific standard.

# Peer review: Reviewer response for version 1

**DOI:** 10.21956/gatesopenres.14705.r32296

**Published:** 2022-08-05

**Authors:** Sylvia K. Ofori

**Affiliations:** 1 Department of Biostatistics, Epidemiology and Environmental Health Sciences, Jiann-Ping Hsu College of Public Health, Georgia Southern University, Statesboro, GA, USA

**Summary:**

 The study uses agent-based modeling to assess the impact of different vaccination programs, specifically Supplementary Immunization Activities (SIA) on COVID-19 in urban, rural-urban jurisdictions and healthcare workers who travel to vaccinate vulnerable children. The authors focused on countries in each of the six WHO regions; Angola, Ecuador, Lao PDR, Nepal, Pakistan, and Ukraine. Adequate background on the impact of the COVID-19 pandemic on SIA and Routine Immunizations was provided by the authors. The study concluded that fixed-post SIAs in urban regions during high SARS-CoV-2 prevalence were associated with an increase in cases.

**General comments:**

- The authors referred readers multiple times to the extended data for certain details (I am assuming this may be due to the word limit). Important details on the epidemiological model are needed in the main text as it is difficult for the reader to appreciate what the authors set out to do.
- The conclusion of the study and recommendations for practice are not clear and do not fully support the objectives of the study (especially for the selected countries, and the implications for other LMICs). I am not sure why the authors highlighted the selection of countries from each of the WHO regions in the analysis as there seem to be no comparisons in the narrative of the results. Also, the objective in the abstract states that the authors conducted the study to inform approaches to resume services, however, the conclusions do not adequately highlight the recommended approaches.
- What were some of the limitations of the model used based on assumptions made by the authors? Any recommendations for future researchers to consider addressing these limitations?
- In the discussion section of the paper, the authors suggested that the role of HW in transmission could be avoided when local HW in rural regions was responsible for administering the vaccines. Did the authors consider exploring a scenario of using only local HW for the house-to-house immunizations and how that could have affected the outcome? Aside from the use of local vaccination staff where possible, are there any practical recommendations to lower the importation risk from HWs?

**Minor comments:**

- In the abstract, is there any reason the authors highlighted results for urban regions and healthcare workers in the results, but the conclusion focused on something else?
- The results for the countries included in the study could also have been highlighted in the results section.
- Some acronyms are not included in the abbreviation list.
- How were countries in each of the six WHO regions selected? Were there any specific criteria, was it random or arbitrary? This is not clear in the main text as the authors only mentioned that a country was selected from each region.
- Any reason for specifically redistributing 20% of reduced contacts to the home route? Was the percentage varied in an uncertainty analysis?
- In the methods, rural-only networks were not considered. Although the authors explained what that network would look like, an explanation as to why this was excluded was not given.
- The circles in figure 2 seem to suggest that the selected countries have a larger proportion of persons <15 years and a rural population.

Is the work clearly and accurately presented and does it cite the current literature?

Partly

If applicable, is the statistical analysis and its interpretation appropriate?

Not applicable

Are all the source data underlying the results available to ensure full reproducibility?

Yes

Is the study design appropriate and is the work technically sound?

Yes

Are the conclusions drawn adequately supported by the results?

Partly

Are sufficient details of methods and analysis provided to allow replication by others?

Partly

Reviewer Expertise:

Infectious disease epidemiology and modeling

I confirm that I have read this submission and believe that I have an appropriate level of expertise to confirm that it is of an acceptable scientific standard, however I have significant reservations, as outlined above.

# Response

**Published:** 2022-09-17

**Authors:** Kurt Frey

**Affiliations:** Bill & Melinda Gates Foundation, USA

**Reviewer 2**

**Authors’ Response**

*Thank you for your time and consideration in reviewing this manuscript. It is greatly appreciated. All feedback has been reproduced below with a detailed reply to each point (indicated using an italic font).*

**Reviewer - Summary**

 The study uses agent-based modeling to assess the impact of different vaccination programs, specifically Supplementary Immunization Activities (SIA) on COVID-19 in urban, rural-urban jurisdictions and healthcare workers who travel to vaccinate vulnerable children. The authors focused on countries in each of the six WHO regions; Angola, Ecuador, Lao PDR, Nepal, Pakistan, and Ukraine. Adequate background on the impact of the COVID-19 pandemic on SIA and Routine Immunizations was provided by the authors. The study concluded that fixed-post SIAs in urban regions during high SARS-CoV-2 prevalence were associated with an increase in cases.

**Reviewer - Comments**

 1. The authors referred readers multiple times to the extended data for certain details (I am assuming this may be due to the word limit). Important details on the epidemiological model are needed in the main text as it is difficult for the reader to appreciate what the authors set out to do.

*This article was originally drafted to respect a 3000-word limit, but there is no restriction on article length at Gates Open Research. More details for the epidemiological model from appendix 1 have been added to the Methods section of the article.*

 2. The conclusion of the study and recommendations for practice are not clear and do not fully support the objectives of the study (especially for the selected countries, and the implications for other LMICs). I am not sure why the authors highlighted the selection of countries from each of the WHO regions in the analysis as there seem to be no comparisons in the narrative of the results. Also, the objective in the abstract states that the authors conducted the study to inform approaches to resume services, however, the conclusions do not adequately highlight the recommended approaches.

*One country was selected from each WHO region to demonstrate that the approach to estimating impact was general to a wide variety of countries. Individual countries were used as examples so that parameter combinations (e.g., age distributions and urbanization) were representative of specific places, in order to inform global guidance. Precise recommendations to modeled countries were not provided because the authors did not have direct familiarity with country-specific policies.*

*The goal of this study was to evaluate the potential range of impact SIAs could have on the novel coronavirus pandemic, and to provide quantitative evidence for in-country decision makers when they were weighing the relative health impacts of suspending SIAs for diseases like measles and polio vs. SARS-CoV-2 risks. We found that SIAs caused modest increases in SARS-CoV-2 infections, which can then be compared to impacts on SIAs’ impacts on other vaccine preventable diseases.*

*Additional emphasis has been added to the discussion section to assist decision makers, but it is not possible to specifically advocate that SIAs not be suspended, given that this paper is focused strictly on quantifying SARS-CoV-2 and does not model other diseases at the same time. This choice was an intentional one because the range of potential comparators is wide and too much to cover in a single paper.*

 3. What were some of the limitations of the model used based on assumptions made by the authors? Any recommendations for future researchers to consider addressing these limitations?

*This model was developed and implemented in May 2020; it assumed re-infection did not occur and ignored the potential for immune evasion through variant strains. Those were reasonable assumptions based on the observed epidemiology at the time, but the pandemic has evolved since then. An updated model would need to account for waning immunity and differences in variant serotypes, although that effect may be minor. For the purposes of infection, recent variant behavior (e.g., Omicron) could be represented as a novel strain encountering a semi-naïve population, partially mitigated by vaccination and previous exposures.*

*Connecting SARS-CoV-2 infections and COVID-19 burden is also a model limitation. The benefits of SIAs are primarily accrued by children under the age of 5, while the consequences of increased transmission intensity are borne in large part by older populations.*

*These limitations are discussed in the manuscript.*

 4. In the discussion section of the paper, the authors suggested that the role of HW in transmission could be avoided when local HW in rural regions was responsible for administering the vaccines. Did the authors consider exploring a scenario of using only local HW for the house-to-house immunizations and how that could have affected the outcome? Aside from the use of local vaccination staff where possible, are there any practical recommendations to lower the importation risk from HWs?

*Scenarios utilizing local HWs were indistinguishable from the base case; no statistically significant increases in infection were identified. Those outcomes (and other effectively null results) were not included in the manuscript to focus on scenarios that resulted in non-zero burden increases.*

*Utilization of personal protective equipment (PPE) and other non-pharmaceutical interventions is expected to significantly lower importation risk due to an infected HW. Previous versions of the manuscript included a presentation of Relative Acquisition / Transmission Rates (RATR)s in HWs that was focused on the use of PPE as a method for reducing HW infection; although that description was omitted due to concerns of imprecise estimates of the effect of PPE on transmission risk. Careful use of PPE could potentially reduce HW risk below that experienced by the public (i.e., RATR values <1.0), although those scenarios were not considered.*

**Reviewer - Other minor comments**

 1. In the abstract, is there any reason the authors highlighted results for urban regions and healthcare workers in the results, but the conclusion focused on something else?

*The original abstract was formatted to respect a 200-word maximum, but there is no restriction on abstract length at Gates Open Research.*

*Additional emphasis on the SIA scenarios has been added to the Conclusions subsection of the abstract.*

 2. The results for the countries included in the study could also have been highlighted in the results section.

*Trends in country specific outcomes have been added to the Results section.*

 3. Some acronyms are not included in the abbreviation list.

*All abbreviations have been added to the Abbreviations section.*

 4. How were countries in each of the six WHO regions selected? Were there any specific criteria, was it random or arbitrary? This is not clear in the main text as the authors only mentioned that a country was selected from each region.

*Appendix 4 in Additional File 1 lists country-level indices for all lower- or middle-income countries with a population of greater than one million. All listed countries were considered for use as representative contexts. Countries were selected to span the range of key indicator values and based on the expertise of our WHO co-authors.*

*To clarify this, the Example Country Selection subsection under Methods has been amended to describe how countries were selected to span the range of indicators.*

 5. Any reason for specifically redistributing 20% of reduced contacts to the home route? Was the percentage varied in an uncertainty analysis?

*Using 20% for redistribution of contacts was a choice made based on assumptions of the effect of behavior modification and was not varied as part of an uncertainty analysis.*

 6. In the methods, rural-only networks were not considered. Although the authors explained what that network would look like, an explanation as to why this was excluded was not given.

*Rural only networks were not considered because individual importations often do not result in community transmission. Forcing importation of virus to a rural community causes behavior analogous to miniature urban environments* *.*

*Description added to the text.*

 7. The circles in figure 2 seem to suggest that the selected countries have a larger proportion of persons <15 years and a rural population.

*We apologize for the confusion. We have noted in the text that size is only intended to highlight countries of interest and their locations.*

# Peer review: Reviewer response for version 1

**DOI:** 10.21956/gatesopenres.14705.r32200

**Published:** 2022-06-24

**Authors:** Sabina Marchetti

**Affiliations:** 1 Bank of Italy, Rome, Italy

The paper provides insights on the trade-off between preventive and routine immunization activities against a number of diseases and SARS-CoV-2 transmission induced by mass vaccination campaigns. Changes in the number of infections derived from individual-based transmission model scenarios with simplified spatial networks providing a stylised representation of plain urban and urban-rural dynamics. The analysis considers one representative low-medium income country for each WHO regions, namely Angola, Ecuador, Lao PDR, Nepal, Pakistan and Ukraine.

**Comments:**

 1. Albeit the authors refer readers to Extended data (and to “additional file 1”, that I could not find..?) for details on the model characteristics, the paper may benefit from high-level descriptions or examples on the main trajectories entailed by the individual-based model. For instance, I found hard to grasp any details on the paths and specificities of health workers within the developed framework, even though the authors underline their key role in SARS-CoV-2 transmission from urban to rural areas for their analysis and policy relevance. Moreover, it should be clarified to which extent the pandemic affects health workers: are they re-allocated to, for instance, COVID-19 related tasks, or do they suspend their activity due to infection? To what extent does the model account for asymptomatic individuals?

 2. Overall, I found difficult to appreciate the policy relevance of the work, due to a number of reasons that expose its soundness to several weaknesses. In detail, if the takeaway message was “policy makers should suspend all SIAs until the peak is over”, it ought to be put in connection with insights on how this would reflect on the different diseases addressed by SIAs. This would enable a comprehensive analysis of the trade-off between SARS-CoV-2 transmission and deployment of SIAs.

 I am under the impression (but I might be wrong and therefore would require clarification by the authors in the text) that the main “policy takeaway messages” rely on a set of assumptions that are hard to validate and prevent straightforward generalisation to any LMIC. For instance, the discussion might support the goodness of suspending SIAs until after the (ascertained) end of the peak, to protect (mainly the elderly?) from SARS-CoV-2. Throughout, SIAs would address (primarily) children who are assumed (but not proved for the ancestral strain of the virus, nor unequivocally for ensuing variants) to be less at risk. Even so, how do the authors compare the quality of life of a non-immunised child via SIA and that of an adult hospitalised due to SARS-CoV-2 infection (i.e. are the authors accounting for medium and long term implications while advising policy makers)? As a comprehensive analysis of the general burden of disease would be out of the scope of the paper, I recommend the authors lay down all caveats so as to clarify the limitations and key assumptions of their framework.

 Along this line, authors might discuss the implications of the absence of a mechanism underlying individual behaviour. While the authors acknowledge self-induced behavioural dynamics plays a key role in outbreaks (in the Methods: Transmission Modelling subsection), their relevance close to a pandemic peak and the documented asymmetry in responsiveness between urban and rural populations might affect the depicted scenarios (e.g., Li *et al*., 2021 ^1^).

**Other minor comments:**

 1. The paper is full with acronyms that are known to many readers or can be drawn from the context. However, authors ought to explicate and/or include them in the abbreviation list. They include - I might have missed others - VPDs, DTP3, EMOD (in this case the citation might be referred to the acronym), LIMC, HW.

 2. The sentence in the Introduction “The WHO […] advised temporary suspension of mass vaccination campaigns based on the recommendations for physical distancing and the understanding of SARS-CoV-2 transmission” seemingly suggests distribution of available vaccines against COVID-19 was suspended upon WHO recommendation whereas it should be clarified it refers to those against diseases different from COVID-19.

 2. In the Introduction, add reference to “WHO regions”, to help the reader.

 3. In the Methods, authors should specify the literature and empirical analyses suggesting children cohorts are characterised by lower susceptibility and transmissibility rates primarily refer to the original strains of the virus, whereas the same cannot be ascertained for successive variants.

 4. Figure 2: Specify size is only intended to highlight annotated dots.

 5. Figure 3: Clarify infection from what in the caption and extend limits of the y-axis, to enable full representation of the curves for Ukraine.

 6. Figure 3/Figure 4: Not sure about what authors are pointing out here: “allowing health workers to administrate SIA within a pandemic peak should be avoided”? How does this compare to “keep some random individuals moving within and between areas at some point around the peak” from the model standpoint? As a further (low-level) observation, annotation of the SARS-CoV-2 incidence peaks on all panels might help the reader.

Is the work clearly and accurately presented and does it cite the current literature?

Partly

If applicable, is the statistical analysis and its interpretation appropriate?

Not applicable

Are all the source data underlying the results available to ensure full reproducibility?

Yes

Is the study design appropriate and is the work technically sound?

Partly

Are the conclusions drawn adequately supported by the results?

No

Are sufficient details of methods and analysis provided to allow replication by others?

No

Reviewer Expertise:

Mathematical modeling of infectious diseases; Statistics

I confirm that I have read this submission and believe that I have an appropriate level of expertise to confirm that it is of an acceptable scientific standard, however I have significant reservations, as outlined above.

## References

[1] : Transmission Dynamics, Heterogeneity and Controllability of SARS-CoV-2: A Rural–Urban Comparison. *International Journal of Environmental Research and Public Health* .2021;18(10) : 10.3390/ijerph18105221 10.3390/ijerph18105221 PMC815672134068947

# Response

**Published:** 2022-09-17

**Authors:** Kurt Frey

**Affiliations:** Bill & Melinda Gates Foundation, USA

**Authors’ Response**

*Thank you for your time and consideration in reviewing this manuscript. It is greatly appreciated. All feedback has been reproduced below with a detailed reply to each point (indicated using italic font).*

**Reviewer - Summary**

 The paper provides insights on the trade-off between preventive and routine immunization activities against a number of diseases and SARS-CoV-2 transmission induced by mass vaccination campaigns. Changes in the number of infections derived from individual-based transmission model scenarios with simplified spatial networks providing a stylised representation of plain urban and urban-rural dynamics. The analysis considers one representative low-medium income country for each WHO regions, namely Angola, Ecuador, Lao PDR, Nepal, Pakistan and Ukraine.

**Reviewer - Comments**

 1. Albeit the authors refer readers to Extended data (and to “additional file 1”, that I could not find..?) for details on the model characteristics, the paper may benefit from high-level descriptions or examples on the main trajectories entailed by the individual-based model. For instance, I found hard to grasp any details on the paths and specificities of health workers within the developed framework, even though the authors underline their key role in SARS-CoV-2 transmission from urban to rural areas for their analysis and policy relevance. Moreover, it should be clarified to which extent the pandemic affects health workers: are they re-allocated to, for instance, COVID-19 related tasks, or do they suspend their activity due to infection? To what extent does the model account for asymptomatic individuals?

*An OSF registry for v01 of the submitted manuscript is located at* https://osf.io/c3dxr *; a direct link to the additional file 1 is* https://osf.io/c3dxr/files/osfstorage/61f5eeeb026ee60528b502ab *.*

*This article was originally drafted to respect a 3000-word limit, but there is no restriction on article length at Gates Open Research. More details for the epidemiological model have been added from appendix 1 to the methods section of the article.*

*In these simulations, health workers are re-allocated to COVID-19 related tasks (SIAs) during task implementation periods but are assumed to be involved in other health-related activities during other periods. A health worker is assumed to be always at or above baseline risk for infection by SARS-CoV-2, as indicated by their relative acquisition/transmission rate (RATR) for a given simulation. Health workers are assumed to self-isolate (given symptoms) at the same rate as non-health workers. This has been clarified in the methods section of the manuscript.*

*For all infections, latent and infectious periods are drawn from appropriate distributions for all simulated individuals, including healthcare workers. All infections have a pre-symptomatic phase, although only about 10% of infections will be totally asymptomatic (totally asymptotic infections are those with infectious periods less than the difference between latent and incubation periods); this outcome occurs because of the epidemiological assumptions about latency and incubation and is not an input to the model, so is not described in the assumptions.*

 2. Overall, I found difficult to appreciate the policy relevance of the work, due to a number of reasons that expose its soundness to several weaknesses. In detail, if the takeaway message was “policy makers should suspend all SIAs until the peak is over”, it ought to be put in connection with insights on how this would reflect on the different diseases addressed by SIAs. This would enable a comprehensive analysis of the trade-off between SARS-CoV-2 transmission and deployment of SIAs.

*This work was not intended as advocacy for suspending SIAs and the results show the opposite. At the time of the investigation (May – Aug 2020), many SIAs were being suspended, and it was the intent of the authors to provide modeling to quantify the SARS-CoV-2 impact if these campaigns were to be run instead. The results are in direct support of continuing and/or resuming SIAs. Specifically, estimates of marginal increases in SARS-CoV-2 infection rates if an SIA were to occur at or around peak SARS-CoV-2 incidence (a plausible worst-case scenario) are low.*

*It wasn’t possible to couple this model with averted burden estimates for the various diseases targeted by SIAs, so the conclusions intentionally avoided specific guidance on SIA implementation decisions and leaves the comparison and decisions about trade-offs to in-country policy makers.*

 I am under the impression (but I might be wrong and therefore would require clarification by the authors in the text) that the main “policy takeaway messages” rely on a set of assumptions that are hard to validate and prevent straightforward generalisation to any LMIC. For instance, the discussion might support the goodness of suspending SIAs until after the (ascertained) end of the peak, to protect (mainly the elderly?) from SARS-CoV-2. Throughout, SIAs would address (primarily) children who are assumed (but not proved for the ancestral strain of the virus, nor unequivocally for ensuing variants) to be less at risk. Even so, how do the authors compare the quality of life of a non-immunised child via SIA and that of an adult hospitalised due to SARS-CoV-2 infection (i.e. are the authors accounting for medium and long term implications while advising policy makers)? As a comprehensive analysis of the general burden of disease would be out of the scope of the paper, I recommend the authors lay down all caveats so as to clarify the limitations and key assumptions of their framework.

*We have intentionally limited the discussion to SARS-CoV-2 infections and do not address COVID-19 burden for the exact reasons expressed by the reviewer. All interventions modeled were targeted to children (<5yrs old), while COVID-19 burden is concentrated in the elderly and other populations with co-morbidities, making the direct comparison of ‘health value’ difficult.*

*Additional emphasis on this limitation has been added to the discussion section.*

 Along this line, authors might discuss the implications of the absence of a mechanism underlying individual behaviour. While the authors acknowledge self-induced behavioural dynamics plays a key role in outbreaks (in the Methods: Transmission Modelling subsection), their relevance close to a pandemic peak and the documented asymmetry in responsiveness between urban and rural

 populations might affect the depicted scenarios (e.g., Li et al., 20211).

*The heterogeneity described in the Li et al. reference is an examination of the overdispersion of transmission (relative to Poisson statistics) observed in urban and rural areas. Specifically, that “the average reproductive number (R) and k [the dispersion parameter] did not [differ between urban and rural areas].” The heterogeneity in this manuscript incorporates uniform reproductive numbers and dispersion parameters, as described by Li et al., along with structural differences to represent the contact pattern variation between urban and rural settings.*

*Additional discussion on the absence of an underlying mechanism for individual behavior has been added.*

**Reviewer - Other minor comments**

 1. The paper is full with acronyms that are known to many readers or can be drawn from the context. However, authors ought to explicate and/or include them in the abbreviation list. They include - I might have missed others - VPDs, DTP3, EMOD (in this case the citation might be referred to the acronym), LIMC, HW.

*Thank you for the list. All abbreviations have been added to the Abbreviations section.*

 2. The sentence in the Introduction “The WHO […] advised temporary suspension of mass vaccination campaigns based on the recommendations for physical distancing and the understanding of SARS-CoV-2 transmission” seemingly suggests distribution of available vaccines against COVID-19 was suspended upon WHO recommendation whereas it should be clarified it refers to those against diseases different from COVID-19.

*This guidance was issued in early 2020, before availability of a safe and effective vaccine against COVID-19. The text has been clarified that the guidance does not pertain to COVID vaccination.*

 3. In the Introduction, add reference to “WHO regions”, to help the reader.

*Noted in the text.*

 4. In the Methods, authors should specify the literature and empirical analyses suggesting children cohorts are characterized by lower susceptibility and transmissibility rates primarily refer to the original strains of the virus, whereas the same cannot be ascertained for successive variants.

*Thank you for this point. We have clarified in the methods section that these estimates are only appropriate for the ancestral strain of SARS-CoV-2 and not the variant lineages.*

 5. Figure 2: Specify size is only intended to highlight annotated dots.

*Thank you, we have noted this in the text.*

 6. Figure 3: Clarify infection from what in the caption and extend limits of the y-axis, to enable full representation of the curves for Ukraine.

*We have clarified the captions for figures 3 and 4 to indicate that these values are for SARS-CoV-2 infections. We have also re-scaled the y-axis in Figure 3 per the reviewer’s suggestion.*

 7. Figure 3/Figure 4: Not sure about what authors are pointing out here: “allowing health workers to administrate SIA within a pandemic peak should be avoided”? How does this compare to “keep some random individuals moving within and between areas at some point around the peak” from the model standpoint? As a further (low-level) observation, annotation of the SARS-CoV-2 incidence peaks on all panels might help the reader.

*The contrast between figures 3 and 4 was intended to highlight the differences between administering an SIA in a well-mixed (i.e., urban) environment and an environment with intermediate connectivity (i.e., urban-rural). In a high connectivity environment, the increased number of infections associated with an SIA is in proportion to the increased contact rate during the SIA, while with intermediate connectivity, there is a possibility of non-linear effects.*

*The implementation of house-to-house vaccination was implemented as a random (contact forming) walk among the target (<5yr old) population by health workers (individuals potentially at elevated risk of being infected/infectious). The intent of the study was to quantify the marginal increase in infections attributable to this activity.*

*Time of peak incidence is noted in Table A7-1. We did not include it in the figure to avoid overcrowding the image.*
